# Prevalence of occult hepatitis B virus infection among patients receiving haemodialysis in Sana'a city

**DOI:** 10.1016/j.ijregi.2022.09.004

**Published:** 2022-09-20

**Authors:** Bodoor Ali Hussen Al-Masoodi, Alariqi Reem, Saleh S. Bahaj, Raja M. Al-Haimi, Hassan A. Al-Shamahy, Nagib Abuasba, Abdu-Raoof Mohammad Al-shawkany, Jay Prakash Prasad Kumal

**Affiliations:** a Medical Laboratory Department, Faculty of Medical Sciences, Sana'a University, Sana`a, Yemen; b College of Veterinary Medicine, Sana`a University, Sana`a, Yemen; c Janaki Medical College and Teaching Hospital, Ramdaiya Bhawadi, Janakpurdham, Nepal

**Keywords:** HBV, Occult hepatitis B virus, Haemodialysis patients, Conventional PCR, Sana'a city

## Abstract

**Background:**

Occult hepatitis B virus infection (OBI) is a challenging entity. Due to the increase in invasive procedures, blood transfusions, and difficulties in diagnosing OBI, patients are more likely to acquire OBI. This cross-sectional study was conducted to assess the prevalence of OBI by hepatitis B virus (HBV)-DNA detection, the prevalence of HBV infection by total hepatitis B core antibody (HBcAb) detection, and the potential risk factors for HBV infection in patients receiving haemodialysis regularly.

**Methods:**

This study included 80 patients receiving haemodialysis regularly, without acute or chronic HBV infection. They were selected from the dialysis units in Sana'a city, Yemen from June 2016 to June 2017. Patients who were positive for hepatitis B surface antigen and hepatitis B surface antibody were excluded from this study. Blood samples were taken prior to each haemodialysis session, and serological markers of HBV were included. HBcAb was measured by enzyme-linked immunosorbent assay, and HBV-DNA was measured by polymerase chain reaction.

**Results:**

HBV-DNA was detected in four patients (5%) and HBcAb was detected in 38 patients (47.5%). There was a significant association between HBV-DNA and HBcAb in patients receiving haemodialysis regularly.

**Conclusions:**

Patients who test positive for HBcAb should undergo additional HBV-DNA testing to allow accurate HBV screening and prevent infection of other patients.

## Highlights

• This study aimed to determine the prevalence of occult hepatitis B virus infection and the prevalence of hepatitis B virus infection among patients receiving haemodialyis in Sana'a, Yemen.
• A significant association was found between total hepatitis B virus core antibody (T-HBcAb) and hepatitis B virus-DNA.
• No significant association was found between positive or negative HBcAb and risk factors.

## Introduction

Hepatitis is a general term indicating hepatic inflammation defined by inflammatory cells. Most cases of hepatitis are caused by a group of viruses known as the hepatitis viruses. Humans are the only reservoir of hepatitis B virus (HBV) (Gerlich et al., 2010). The incidence of HBV infection varies widely by geographic area. It is estimated that 350 million people are affected by HBV worldwide, which corresponds to a high rate of carriage of hepatitis B surface antigen (HBsAg) (up to 20%), particularly in South and East Asia (Allain, 2004). Previous data showed that hepatitis B is hyperendemic in Yemen, and infection with HBV and hepatitis C virus (HCV) is an important cause of chronic liver disease (Murad et al., 2013). Patients receiving haemodialysis have a higher risk of viral hepatitis, and viral hepatitis accounts for 1.9% of deaths (Abdel-Maksoud et al., 2019). HBV is a double-stranded DNA virus from the family Hepadnaviridae and the genus Orthohepadnavirus (Datta et al., 2012). There are three types of HBV infection: acute, chronic and occult (Nakamoto et al., 2014). Occult HBV infection (OBI) is characterized by the presence of hepatitis B virus deoxyribonucleic acid (HBV-DNA) in serum, or on liver biopsy in HBsAg- negative patients with or without previous serological markers virus exposure like (HBsAg), anti-hepatitis B core IgM antibody (anti-HBcIgM) and hepatitis B “e” antigen (HBeAg) (Coppola et al., 2015). HBV is highly contagious, with up to 30% chance of seroconversion. In contrast, the probabilities of infection with HCV and human immunodeficiency virus are 1.8% and 0.31%, respectively. Patients undergoing regular haemodialysis are at increased risk of contracting diseases via the parenteral route as they are immunosuppressed, undergo invasive treatment, respond poorly to the HBV vaccine, and require multiple blood transfusions, and also due to the duration of dialysis. The prevalence of OBI is unknown and is determined by the sensitivity of the HBsAg and DNA assays used. Endemic HBV infection is also associated with OBI. Patients from areas where HBV is prevalent are more likely to develop OBI (Onorato et al., 2021). OBI can accelerate the progression of cirrhosis and hepatocellular cancer (Squadrito et al., 2014). If cirrhosis and hepatocellular carcinoma develop, reactivation occurs, leading to acute hepatitis which can be fatal (Song and Hwang, 2013). This study investigated patients receiving haemodialysis regularly in Sana'a city, Yemen in order to determine the prevalence of OBI by detecting HBV-DNA using conventional polymerase chain reaction (PCR), and to determine the prevalence of HBV infection by detecting hepatitis B core antibody (HBcAb).

## Materials and methods

### Subjects

This cross-sectional study was conducted on 80 patients with renal failure in haemodialysis units at Al Thawra Modern General Hospital and Al Jamhouri Teaching Hospital, Sana'a city from June 2016 to June 2017. Data on the medical history of each patient, including name, age, sex, address and duration of haemodialysis, were collected using a questionnaire. Patients who were HCV positive and HBsAg positive were excluded.

### Specimen collection

Five millilitres of venous blood were drawn from each patient in the haemodialysis sessions, and the samples were divided into two parts, each labelled with patient name and serial number. The first part consisted of serum in a vacuum tube, which was separated from whole blood after being allowed to clot for 15 min, followed by centrifugation at 3000 rpm for 10 min. Thereafter, it was transferred to Eppendorf tubes with a pipette to detect HBsAg, HBsAb and total (T)-HBcAb. The second part consisted of 2 mL of whole blood placed in an EDTA tube for detection of HBV-DNA by conventional PCR.

### Determination of HBsAg, HBsAb and T-HBcAb by ELISA

Enzyme-linked immunosorbent assays (ELISA) for HBsAg, HBsAb and T-HBcAb were performed in accordance with the test kits (ARCHITECT i 1000 SR System, Abbott Diagnostics, Abbott Park, IL, USA). HBsAg-positive samples were excluded from the study (patients receiving haemodialysis were selected according to the dialysis record in which they were HBsAg, HBsAb negative), and every patient seprated from the other in the dialysis unite (Martinez et al., 2015).

### Determination of HBV-DNA by conventional PCR

#### DNA extraction from whole blood

The AccuPrep Genomic DNA Extraction Kit (Cat. No: K-3032; Bioneer, Daejeon, South Korea) was used to extract DNA from all whole blood samples in accordance with the manufacturer's instructions (Kchour et al., 2017).

#### PCR amplification

The HbsAg gene was amplified using forward primer P1 (nt 2823- 2845; 5´-TCA CCA TAT TCT TGG GAA CAA GA-3′) and reverse primer S1-2 (nt 685-704; 5´-CGA ACC ACT GAA CAA ATG GC-3′). For amplification, initial pre-denaturation was performed at 95°C for 5 min, followed by denaturation at 95°C for 30 s, primer annealing at 57°C for 30 s, elongation at 68°C for 80 s, and final extension at 68°C for 5 min. The sample was stored at -20°C until the following day for analysis. Template DNA and primers were added to the AccuP tubes of the reaction mixture (DNA template 5 µL, forward primer 0.5 µL, reverse primer 0.5 µL, sample DNA 5 µL and PCR-grade water 14 µL). The lyophilized blue pellet dissolved completely, and was centrifuged with ExiSpin Vortex/Centrifuge for 15 s at high speed, followed by 5-s spins at 1500 rpm for four cycles (AccuPower ProFi Taq PCR PreMix, South Korea) (Rashid and Salih, 2015).

#### Electrophoresis of the amplified product

**Figure 1:**
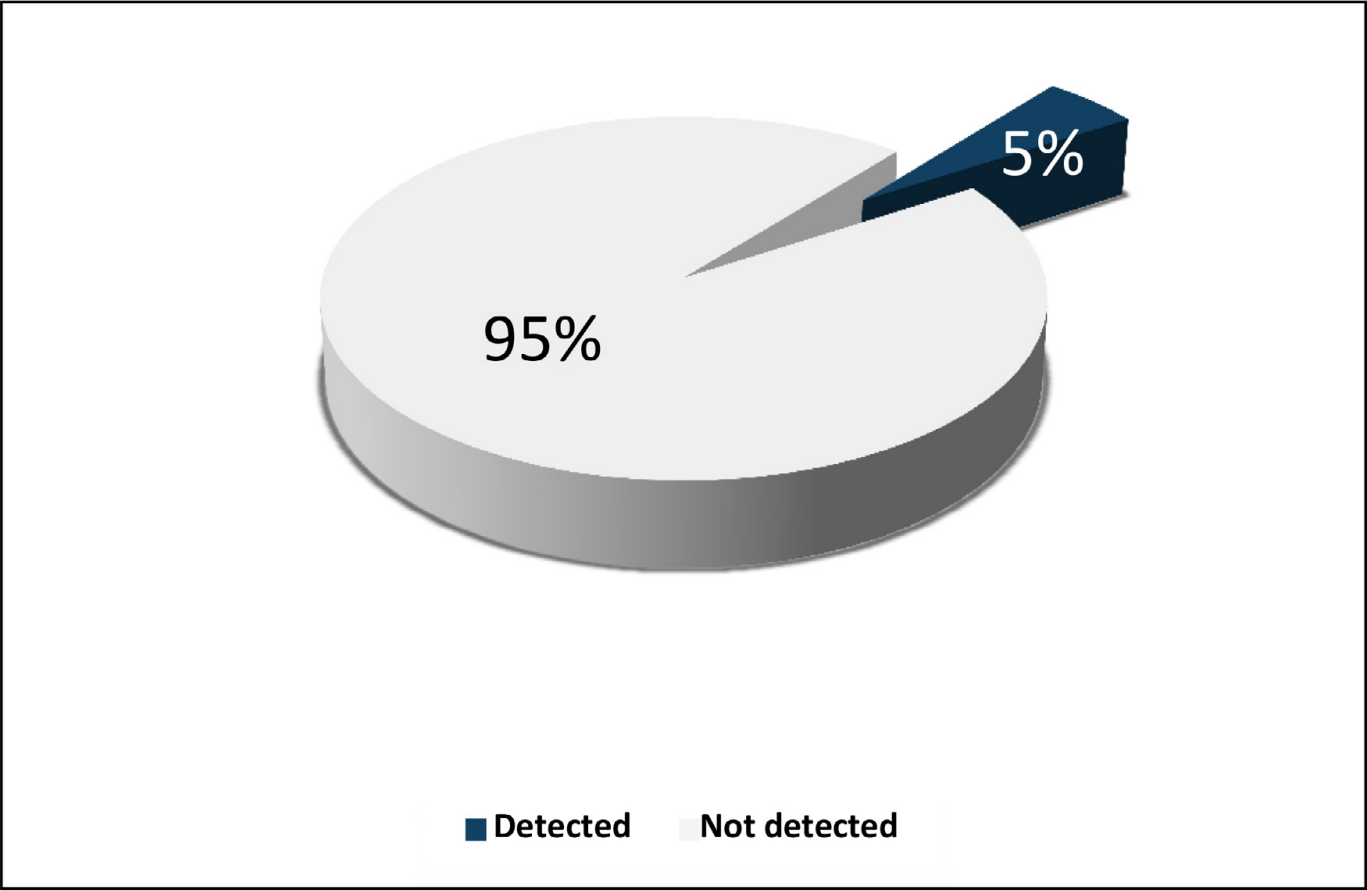
Electrophoresis of hepatitis B virus-DNA. Lane 1, ladder (100-500 +1000+1500+3000 bp); lane 2, positive control, lane 3, negative control; lanes 4, 5, 6 and 7, positive samples.

The PCR products were analysed by gel electrophoresis in 2% agarose gels in TBE buffer. Gels were run at 100 V for 40 min in 1x TAE containing 1 µL ethidium bromide. The PCR products were visualized with ultraviolet light (Figure 1). The bands were aligned at 1063 base pairs (bp) indicating a positive reaction (Rashid and Salih, 2015).

### Statistical analysis

Collected data were encrypted and entered into SPSS Version 21 (IBM Corp., Armonk, NY, USA). Quantitative data were plotted as mean and standard deviation. The percentage of variables for each category provided was quantified. Statistical comparison between categorical variables was performed using Chi-squared pair test, and *P*≤0.05 was considered to indicate significance.

## Results

**Figure 2:**
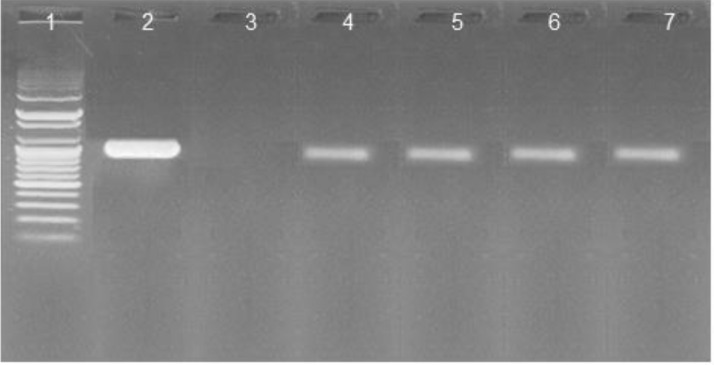
Prevalence of occult hepatitis B virus infection among patients in haemodialysis units in Sana'a city, 2016.

The sociodemographic variables of patients receiving haemodialysis are summarized in Table 1. Among the 80 patients, 38.8% (*n*=31) were aged 20–40 years, 37.5% (*n*=30) were aged 41–60 years, 17.5% (*n*=14) were aged >60 years, and 6.2% (*n*=5) were aged <20 years. Overall, 53.8% (*n*=43) of patients were male and 46.2% (*n*=37) were female.

a- *In terms of the prevalence of OBI, four of the eighty patients (5%) were HBV-DNA positive, and 76 (95%) were HBV-DNA negative (*Figure 2*).*

**Table 1:** Demographic characteristics of patients receiving haemodialysis in Sana'a city, 2016.

Demographic characteristics		*n*	%
		35.5±10.4^a^	
Age group (years)	< 20	5	6.2
	20–40	31	38.8
	41–60	30	37.5
	>60	14	17.5
	Total	80	100
Sex	Male	43	53.8
	Female	37	46.2
	Total	80	100

a Mean ± standard deviation.

The prevalence of OBI in patients receiving haemodialysis was 10.5% (*n*=4). None of these four patients had a negative HBcAb value. An association was found between the presence of HBV-DNA and T-HBcAb (Table 2).

**Table 2:** Association of hepatitis B virus (HBV)-DNA with total hepatitis B core antibody (T-HBcAb) in patients receiving haemodialysis in Sana'a city, 2016.

Characteristics		HBcAb						χ^2^^a^	OR	CI		*P-*value^b^
		Positive		Negative		Total						
		*n*	%	*n*	%	*n*	%			Lower	Upper	
HBV-DNA assay	Detected	4	10.5	0	0	4	5					
	Not detected	34	89.5	42	100	76	95	4.65	2.23	1.74	2.87	0.047
	Total	38	47.5	42	52.5	80	100					

OR, odds ratio; CI, confidence interval.

a Fisher's exact test, χ^2^≥3.9.

b *P*≤0.05 was considered to indicate significance.

Table 3 shows the risk factors for T-HBcAb and HBV-DNA infection in patients receiving haemodialysis. In patients receiving dialysis outside of what was specified (100%), surgical history (66.7%) with positive T-HBcAb, blood transfusion (54.2%) with positive T-HBcAb, and specified dialysis for 1 year (50%) and 1 year (16.7%) in positive T-HBcAb, the converged proportions were longer (Taha et al., 2013). They were found in patients with mix factors (50%) in HBcAb who had undergone dental treatments and had positive T-HBcAb (0%). While there were patients in family history of HBV, the HBcAb is (0%), and all risk factors of this study are statistically insignificant. As the abovementioned methods were used to spread HBV, they have been recognized as risk factors.

**Table 3:** Potential risk factors for contracting hepatitis B core antibody (HBcAb) in patients receiving haemodialysis in Sana'a, 2016.

Characteristics		HBcAb positive		HBcAb negative		χ^2^	OR	CI		*P-*value^b^
		*n*	%	*n*	%			Lower	Upper	
Duration of dialysis (years)	<1	1	16.7	5	83.3	2.47^a^	0.20	0.22	1.79	0.20
	≥1	37	50	37	50					
Blood transfusion	Yes	13	54.2	11	45.8	0.61	1.46	0.56	3.82	0.47
	No	25	44.6	31	55.4					
Surgical history	Yes	2	66.7	1	33.3	0.62^a^	2.27	0.19	26.1	0.60
	No	36	46.8	41	53.2					
Dental procedure	Yes	0	0	4	100	0.38^a^	0.20	1.58	2.50	0.11
	No	38	50	38	50					
Dialysis outside specified the aimes of the study	Yes	1	100	0	0	1.16^a^	2.13	1.68	2.70	0.47
	No	37	46.8	42	53.2					
Family history	Yes	0	0	0	0	Undefined				
	No	38	47.7	42	52.5					
Mix factors	Yes	21	50	21	50	0.55	1.11	0.46	2.67	0.82
	No	17	44.7	21	55.3					

OR, odds ratio; CI, confidence interval.

a Fisher's exact test, χ^2^≥3.9.

b *P*≤0.05 was considered to indicate significance.

## Discussion

OBI is receiving more attention due to its clinical relevance in the development of chronic liver disease, and its relationship with hepatocellular carcinoma is growing (Taha et al., 2013). Due to frequent invasive procedures, prolonged vascular access with high exposure to infected patients and contaminated equipment, a higher number of blood transfusions, a longer duration of dialysis and immunosuppression, patients receiving haemodialysis regularly are more likely to acquire OBI. Than other patient who dont do the OBI test. Liver biopsies of patients receiving haemodialysis tend to be difficult to perform, and are not usually recommended. Therefore, most OBI diagnoses are based on blood test results (El-Ghitany et al., 2013). The prevalence of OBI ranges from <1% to 87% in patients in dialysis units. Due to the differing prevalence of HBV infection, and differing sensitivity and specificity of detection methods, the available data show that its prevalence of HBV infection varies between countries (Sharifi-Mood et al., 2009). To the authors’ knowledge, in Yemen, only one study has been published on the number of people who had OBI with chronic liver disease, and the prevalence rate was 4.3% (Thabit et al., 2012). The present study is the first study to address this issue in the dialysis units of Sana'a city, and aimed to determine: (1) the prevalence of OBI in patients receiving haemodialysis by detecting HBV-DNA using a conventional PCR technique; (2) the prevalence of HBV infection in patients receiving haemodialysis by detecting T-HBcAb; and (3) the potential risk factors for HBV infection in patients receiving haemodialysis. Based on the detection of HBV-DNA, which is the gold standard test for OBI, this study found that the prevalence of OBI in patients receiving haemodialysis was 5%. This result is consistent with the rates in Western and Upper Egypt (6% and 4.1%, respectively) (Bedewy and Ibrahim, 2006), but higher than the rates in Tehran, Iran (1%) (Ramezani et al., 2015), Hong Kong (1.1%) (Tang et al., 2016) and China (1.3%) (Kim et al., 2015). Other studies, including one in Alexandria, Egypt, have also found a high incidence of OBI (32%) (Helaly et al., 2015). These differences in the OBI rate among patients receiving haemodialysis, and differences in studies reporting conflicting results in the same country, could be related to differences in the prevalence of HBV infection in different countries. The adoption of safety precautions in dialysis units and the implementation of routine immunization programmes are other differences that have been studied for various molecular biological methods for the detection of HBV-DNA (Polaris Observatory Collaborators, 2018).

This study found that the prevalence of T-HBcAb was 47.5% in the 80 patients who tested negative for HBsAg on routine ELISA, which is consistent with a previous report from northeastern Brazil (38%) (Fontenele et al., 2015). Other studies have reported differential prevalence rates of HBcAb in patients receiving haemodialysis in Bushehr province, Iran (6.7%) and in Isfahan, Iran (6.2%) (Mostaghni et al., 2011; Kalantari et al., 2016). These differences could be due to differences in the prevalence of HBcAb in countries. Yemen is categorized as having an intermediate HBV endemicity (Al-Nwany et al., 2021). Most studies with similar results were also conducted in countries with intermediate endemicity for HBV. The highest prevalence rates have been found in countries with moderate or high endemicity for HBV (Zampino et al., 2015). Various serological methods and kits can be used to assess and detect HBV infection. This study found a significant association between HBV-DNA and HBcAb in patients receiving haemodialysis. This result was similar to a study undertaken in Giza, Egypt (Elbahrawy et al., 2015). The results can be explained by the small sample size. Mutated virus cannot be detected with commercially available kits. Differing levels of HBV viraemia during disease between patients could be the reason. The present study did not find an association between positive or negative HBcAb and the following risk factors: duration of dialysis, blood transfusion, surgical history, dental procedure, dialysis outside specified the aimes of the study, and family history of HBV infection. Significant relationships were found between HBV-DNA and HBcAb positivity, and between HBV-DNA and HBeAg positivity. These results can be explained by the small size of the study groups, and few studies examining the impact of risk factors in patients receiving haemodialysis.

### Study limitations

This study had a few limitations. The patients were all from one governorate, private hospitals were not included, and HBV was the only virus studied due to the high financial cost of the tests.

## Conclusions

In conclusion, the prevalence of OBI, based on conventional PCR, was 5% among patients receiving haemodialysis in Sana'a city, Yemen. The prevalence of HBV infection, based on detection of T-HBcAb, in patients receiving haemodialysis in Sana'a city was 47.5%. A significant association was found between T-HBcAb and HBV-DNA. No significant association was found between positive or negative HBcAb and risk factors.

## Conflict of interest statement

None declared.

## Appendix Supplementary materials

Image, application 1
