# Supplementary material for: Prevalence of occult hepatitis B virus infection among patients receiving haemodialysis in Sana'a city
Source: IJID Reg. 2022 Sep 20;5:141–5. doi: 10.1016/j.ijregi.2022.09.004 (PMC9634008; doi:10.1016/j.ijregi.2022.09.004)
Supplement: Supplementary file 1 [file mmc1.docx]

## AUTHORSHIP AGREEMENT

All requests for authorship changes after submission of a manuscript to *IJREGI* should be made to the Editorial Office. This form must be completed for any authorship changes including adding new authors, removing existing authors, reordering existing authors, or adjusting equal contributor status. The corresponding author must confirm that all authors meet the criteria for authorship as outlined by the Committee on Publication Ethics (COPE) and that all authors agree to the change. All authors who have been added, removed, or reordered need to confirm that they agree to the change by signing the form. Please complete and return this form. The relevant submission will be put on hold and no further processing can occur until the editorial office receives the completed form.

## Manuscript number: Manuscript title:

**Would you like to change the authorship of your paper? (**Please tick one of the following):

✓Yes (please fill in box below with all necessary signatures):

**The nature of change(s) in authorship** (Please check one of the following):

- Change de Corresponding Author:

✓Add new author(s);

- Remove existing author(s);
- Change the order of authorship;
- Others (please specify):

## Detailed reason for the change:

The authors add new authors for revision and improve of the manuscript

**Indicate the specific change: Adding a new author**

| **Name** | Abdu-Raoof Mohammad Al-shawkany |
| --- | --- |
| **Email address** | abdualraufe@gmail.com |
| **Institution** | Sana'a University |
| **Specific contribution** | revision and improve of the manuscript |
| **Name** | Jay Prakash Prasad Kumal |
| **Email address** | jppkumal@gmail.com |
| **Institution** | Janaki Medical College |
| **Specific contribution** | revision and improve of the manuscript |

**Removing an existing author**

| **Name** | |  | |
| --- | --- | --- | --- |
| **Email address** | |  | |
| **Institution** | |  | |
| **Agree to be acknowledged** | |  | |

**Complete author order AFTER change (please note any authors with equal contribution as first authors or corresponding authors)**

| **Order** | **Author name BEFORE change** | **Author name AFTER change** | **Signature** |
| --- | --- | --- | --- |
| **1** | Bodoor Ali Hussen Al-Masoodi |  | Bodoor |
| **2** | Alariqi Reem |  | Reem |
| **3** | Saleh S. Bahaj |  | Saleh |
| **4** | Raja M. Al-Haimi |  | Raja |
| **5** | Hassan A. Al-Shamahy |  | Hassan |
| **6** | Nagib Abuasba |  | Nagib |
| **7** | Abdu-Raoof Mohammad Al-awkanyshawkawkany Al-shawkany |  | Abdu-Raoof |
| **8** | Jay Prakash Prasad Kumal |  | Jay |
| **9** |  |  |  |
| **10** |  |  |  |

Approval of the final version of the manuscript to be submitted, all authors must sign the table above to indicate agreement with the altered authorship (all authors must be listed, full name in print and signatures are needed from all).
